# London Letter

**Published:** 1879-08

**Authors:** C. T. Parkes

**Affiliations:** London


### Foreign Correspondence.

---

ARTICLE X.

LONDON, May 28, 1879.

After many years of carefully studied experience in the treatment of almost innumerable cases of hip-joint disease, a large portion of the very best and most erudite surgeons of London, look upon a *good* result as the sequence of excision of the head of the femur, as by far the exception and not at all the rule. Some do not perform the operation, depending upon rest in bed, constant extension, good food and correction of deformity by graduated pressure, to give better and more permanent recoveries than any resort to the knife. These men have done the cutting operation time and time again in their earlier experience, but the results faithfully interpreted and honestly acknowledged, deduced from the records of extensive hospital practice, and unfortunately numerous post mortem examinations, have led them to avoid active interference in almost every case; making it absolutely the *dernier ressort* and then accepting the necessity with faint hearts and doubting minds as to the good done.

Now if there is one crop of maladies which is reaped with an abundant harvest in the special hospitals for children and in general hospitals as well, in London, it is acute and chronic joint disease, especially the latter; strumous if you prefer, although I noted well the particular care with which each surgeon sought for and elucidated the probable traumatic cause or incident which may have preceded the commencement of the incipient trouble, ending in the chronic difficulty presented for examination. In every instance preference was given to the traumatism as compared with constitutional taint, although the latter was not denied a place in the list of causes, nor were its hurtful tendencies overlooked in the matter of predisposition to bad consequences after even very slight injuries. What has been said so far by no means applies to all the surgeons here, nor in any way does it represent the practice adopted in all the hospitals. On the contrary, no joints are left untouched in some. Hip joint disease in these latter means excision, and I am almost justified in saying that the same rule is followed and adopted as the proper treatment in chronic pulpy degeneration of any and all joints in which the operation is at all warrantable. These two courses of procedure are fully represented by the practice of the two largest, wealthiest, most renowned and best patronized hospitals in the city, under the charge of men whose reputations are world-renowned, whose excellence is unsurpassed. It is impossible to explain this belief in opposites, for nothing can be further apart than their plans of treatment, no deductions more widely opposed than are here drawn from the same data, derived from a similar class of cases, by men whose honesty of thought and action impresses the listener, whose knowledge and skill call forth his highest praise, whose charitable sympathy for the sufferer awakens his warmest admiration.

One is impressed with the thought that there must be a middle ground where the greatest safety lies. Perhaps one case witnessed (which is readily recalled) will illustrate the probable existence of this haven of refuge.

A little girl of about eight years of age, had been suffering with all the signs of hip disease for several months, and the stage of suppuration had been reached as evidenced by the sense of fluctuation. She fell into the hands of one who believes and practices excision. The operation was done, and after the separation of the excised head and trochanters, the specimen was immediately sawn through longitudinally by the pathologist who is always present at the operations. The divided bone told the cause of all the trouble at a glance. In the very center of the cancellated tissue of the head lay a sequestrum of dead bone, far removed from any possible chance of exit, and communicating with the "outer world," by the merest thread of a sinus. Nature could have labored for years at its removal and then have fallen short of the completion of the task, for all around this center of months of suffering and bodily decay, was a thick wall of normal, healthy bone. If ever surgical operation was justifiable, this one was—every stroke of the knife but opened the way to health and recovery. It is impossible to contemplate, what consequences would have followed in the wake of the waiting policy, without a shudder. The operation should be done, must be done, often—it is often done when early death or subsequent months and years of open sinuses and prolonged suppuration, convince one that rest, extension and good nourishment would have wrought better results. The choice must be made whatever be the subsequent history. The results following both methods, as here seen, are truly astonishing. I must admit that of the two, my preference now would be to assign by far the greater number of cases to non-operative treatment.

Through the great courtesy of Mr. Howard Marsh, Assistant Surgeon at St. Bartholomew's, it was my pleasure to visit the Hospital for Hip-joint Disease, under his charge, and superintended by charitable ladies. There were some fifty little patients showing all varieties of the disease in all possible stages, accompanied with every kind of deformity—in some both hip-joints were affected, and had added thereto Pott's disease of the spine. All cases which have been discharged from other hospitals as incurable, are admitted here, and one of their absolute rules is, that no cutting operation whatever shall be done—no operation at all other than aspiration of an abcess, if that may be so classed. Each patient has his or her own little cot—they are placed thereon as soon as admitted, flat upon the back; extension with weight and pulley is immediately applied and kept constantly in action. The direction of the extension always corresponds to the line of deformity present in the limb—no forcible attempt is ever made to straighten the limb. The straightening process is a very gradual and easy one indeed, and is secured by means of a movable slide on the frame, upon which the pulley is fastened. It is rather an amusing as well as instructive and impressive sight to see the little legs cocked up at all possible angles all over the room. Experience has taught them that with the adoption of this plan came greater ease to their patients and more rapid recoveries. No complaints are ever heard from the little ones after the first day or two expended in getting used to the newness of the position, unless accidental complications arise. Absolute rest *upon the back* is insisted upon and secured by constant watchfulness, and, better still, by a broad strap of webbing fastened to the bed, to which the shoulders of the patient are tied, so that, if the unruly bump is too well developed and the child attempts to get up as soon as the nurse's back is turned, he soon learns that all attempts result only in discomfiture. Do these children — many in a single room — grow pale and puny under all this restraint and confinement? Not at all, they grow fat and ruddy, their pain is relieved, their sufferings assuaged; kind hands tend to every want; they are, many of them, in better homes than they ever before dreamed of, and better than all, their bellies are filled with the fat of old England. I could not help saying to the doctor and attendants, that the most of the patients did not have any of the general characteristics of children suffering with hip-joint disease, they looked too well, their cheeks were too plump, their lips too red.

Some of the cases of recovery, in good condition after years of treatment commenced, when they were almost in the jaws of death, both hips diseased, almost surpass belief, and would give a thoughtful mien to the most devout believer in the triumphs of the knife.

After the flexion of the limb is overcome, abduction or adduction is counteracted by fixing the hips by a long splint to the sound side, and then a resort to lateral extension by weight and pulley upon the faulty limb and restoration to proper position gradually secured. After the case has gone to the end of this treatment and is practically well, usually with anchylosis, a splint termed Thomas' is fitted to the diseased side, crutches furnished and the little one turned out doors or into the play-room. At the same time the sole of the boot on the sound foot is raised two inches, so that the unsound limb can swing free from the ground and the joint be relieved from sudden jars too early after beginning to use it. Thomas' splint is an iron bar, about five Ctm. wide, and two Mm. thick, extending from well up on the back to the ankle, and bent in curves to fit accurately every curve of the body and limb. Of course it is well padded, and has fitted to it, cushioned side pieces of soft leather, by which it is held in contact with every part of the body and limb. All surgeons here use it, and think well of it. I am positively assured by every one connected with this little hospital, that many permanent recoveries have crowned their efforts at relief, and deaths are far less frequent than after operations.

Where the operative procedure is carried out, the after treatment is quite similar to our own, slight extension. Some do the operation under the carbolized spray. Mr. Lister always, of course. In every operation that I have seen done, the surgeon, after clearing the head of the bone by the usual incision and dissection, has always made his section with the saw, previous to making any attempts to get the head of the bone out of the acetabulum. All seem to think it the better plan of procedure. No one ever claims to obtain a result which gives a more than fairly serviceable limb. The resected head is never entirely restored, no matter what the after treatment, nor do the movements promisable after operation ever equal the normal ones.

The advisability of excision of the knee for chronic degeneration would force into ranks exactly the same opinions and men as we have found opposing each other in questions referring to the hip joint.. The principal opponents to this operation are among men who ten years ago performed the operation often, especially in young patients suffering from progressive joint disease. I mean progressively chronic ; it is to be presumed that no surgeon ever resects an acutely diseased knee joint. The mortality after the operation was so enormous, the good done was so slight in many cases, that they had been forced to believe the only proper course to pursue was narrowed down to one of two; either treat by absolute rest and extension, or amputate. Some of these gentlemen unhesitatingly prefer amputation ; the recovery is rapid, suffering done away with, and the artificial appliances used subsequently, equal in efficacy the limb oftenest left after an excision, after which the agony is many times increased, and death frequently the result. The friends of the operation are equally as sanguine in favor of its benefits. Cases of this chronic nature are fearfully common in London, and it is merely a question of which hospital they enter whether the excision is done or not. A dozen or so have been done during my sojourn here.

In most of them the synovial membrane was much thickened and softened, ulceration of the cartilages was commencing, or had proceeded to advanced destruction. The fluid was either pus, semi-purulent or quite natural. The contour of the joint was destroyed. Palpation gave the usual quasi-fluctuating, semi-elastic feeling. Constitutional depression was gradually becoming more noticeable, and all local and general symptoms showed the disease to be rapidly gaining ground in spite of any and all treatment. It requires a brave heart to be patiently quiescent as such a history gradually unfolds itself to the point where sacrifice of the limb becomes a necessity, or death ends the battle; it takes a bold one to assume the responsibilities of excision. Excisions of the hip joint are often done here as soon as the surgeon by manipulation is fully convinced that the slightest roughness of the cartilaginous surfaces is present, not waiting for the suppurative process, or later stages of the disease. The aim is to anticipate and avoid the suffering and distress which it is claimed surely follows after the trouble has fairly commenced. Such unbounded faith in operative procedure is certainly praiseworthy, if it is only supported by correspondingly good results. We hope to see soon a full statement of the many cases operated upon by those who act upon this faith and belief in treating affections of the hip and knee joint. In this way only can the profession at large draw any conclusions from the practice of equally good men, some of whom operate perhaps two or three times a year while the others see occasion to use the knife almost every week.

C. T. PARKES.

